# Sarcoidosis-Like Reaction in Melanoma Patients Receiving Immunotherapy or Targeted Therapy

**DOI:** 10.1155/crom/8947861

**Published:** 2024-12-16

**Authors:** Giulia Murgia, Luca Valtellini, Gianluca Nazzaro, Emanuela Passoni, Federica Scarfì, Francesca Boggio, Angelo V. Marzano, Ornella Garrone, Nerina Denaro

**Affiliations:** ^1^Dermatology Unit, Fondazione IRCCS Ca' Granda Ospedale Maggiore Policlinico, Milan, Italy; ^2^UOSD Dermatology, USL Toscana Centro-Prato Hospital, Prato, Italy; ^3^Pathology Unit Fondazione, IRCCS Ca' Granda Ospedale Maggiore Policlinico, Milan, Italy; ^4^Oncology Unit Fondazione, IRCCS Ca' Granda Ospedale Maggiore Policlinico, Milan, Italy

## Abstract

This case series highlights the complexity of sarcoidosis-like reactions (SLRs) during cancer treatment, specifically in patients receiving immunotherapy or targeted therapies for melanoma. SLRs can either mimic disease progression or present as part of the clinical manifestation, making diagnosis and treatment challenging. Our study reviewed the medical records of 31 patients who were candidates for postoperative treatment between June 2022 and June 2024. Out of these, three patients developed SLRs during their treatment. A 55-year-old woman with Stage IIIb cutaneous melanoma, receiving adjuvant therapy with anti-PD-1, after seven cycles of pembrolizumab, developed mediastinal node enlargement and skin hypodermic nodes. A biopsy of the hypodermic node revealed granulomatous infiltrates with sparse lymphocytes, consistent with sarcoidosis. A low dose of steroids was administered, resulting in a dramatic improvement. A 48-year-old woman with Stage IIIb BRAF wild-type melanoma, receiving nivolumab every 4 weeks, developed systemic sarcoidosis after seven cycles, primarily affecting extrapulmonary sites. Despite the immune-induced sarcoidosis, her treatment was not stopped, as decided by the multidisciplinary team (MDT). A 65-year-old man with Stage IIIb BRAF-mutant melanoma, receiving dabrafenib and trametinib, developed lung and cutaneous sarcoidosis, presenting with symptoms that led to emergency department admission. In all cases, the MDT played a crucial role in determining the course of treatment and balancing the risks of continuing or suspending cancer therapies while managing SLRs. National and international guidelines were consulted, but tailored decisions by the MDT were essential for optimizing patient care.

## 1. Introduction

Melanoma constitutes about 20% of all skin cancers but is associated with a poor prognosis in its late stages. Originating from melanocytes, melanoma can arise de novo or from pre-existing nevi that undergo malignant transformation. The metastasis of melanoma primarily occurs through the lymphatic system, making lymph node enlargement a critical diagnostic challenge. In later stages, melanoma can spread through the blood, complicating its management.

Despite significant advancements with immune checkpoint inhibitors (ICIs)—such as progressive disease (PD)-1 and CTLA-4 antibodies, which have improved outcomes in metastatic melanoma—the prognosis for advanced-stage melanoma remains generally poor. These therapies, however, have marked a significant breakthrough by enhancing immune responses against the tumor.

Up to 60% of melanomas demonstrate mutations in the BRAF gene, with BRAF V600E being the most common. The BRAF oncogene encodes a protein involved in cellular proliferation, and its mutation leads to uncontrolled growth, making it a key target in therapy. Treatment strategies for melanoma depend on the BRAF mutation status and staging. BRAF inhibitors (often combined with MEK inhibitors) are reserved for patients with Stage III–IV disease. Adjuvant therapy (postsurgical) is recommended for Stage IIb–c melanoma patients, utilizing immunotherapy only. For Stage IIIa–c and Stage IV melanoma patients who are NED (no evidence of disease after radical resection), adjuvant treatments vary. BRAF wild-type patients receive immunotherapy, while BRAF-mutant patients may receive either BRAF/MEK inhibitor therapy or immunotherapy (depending on the disease stage). These tailored therapeutic strategies aim to delay recurrence and improve survival rates in melanoma patients, particularly those with advanced disease.

According to the European Society of Medical Oncology (ESMO), the management of immune-related adverse events (irAEs) follows a structured approach consisting of four key steps: (i) diagnosis and grading of irAEs to assess severity, (ii) ruling out differential diagnoses and performing a work-up before immunosuppression, (iii) selecting an appropriate immunosuppression strategy for irAEs of grade ≥ 2, and (iv) active evaluation at 72 h to adjust the treatment as needed [1].

Sarcoidosis, a systemic disorder of unknown origin, is characterized by granulomas in multiple organs and can either resolve on its own or progress to fibrosis. Pulmonary involvement is observed in 90% of cases, with common systemic symptoms like fatigue, night sweats, cough, and weight loss. The association between sarcoidosis and cancer was first recognized more than 50 years ago [2]. Melanoma patients are particularly susceptible to developing sarcoidosis-like reactions (SLRs), whether undergoing immunotherapy, targeted therapy, or even without receiving any cancer treatment [3]. These SLRs, seen in melanoma, have been correlated with patient outcomes [4]. In some cases, sarcoidosis and cancer have been diagnosed simultaneously or within a short period of one another [4].

The occurrence of SLRs in melanoma patients ranges from 4% to 10%. These reactions typically involve mediastinal lymph nodes and skin, presenting with papules, nodules, or erythematous lesions. Such skin manifestations can easily be mistaken for melanoma metastases, making an accurate diagnosis challenging [1]. These SLRs are clinically indistinguishable from primitive sarcoidosis and occur in close temporal association with the start of an offending drug, typically checkpoint inhibitors like PD-1 or BRAF-targeted therapies.

A combined approach using PET-CT and thallium-201 scintigraphy has been shown to effectively identify systemic sarcoidosis [5].

Sarcoidosis can be triggered by treatments such as ICIs, BRAF/MEK inhibitors, and tumor necrosis factor-alpha (TNF-*α*) blockers. Real-world data indicates that sarcoidosis caused by immune checkpoints or BRAF/MEK inhibitors occurs more frequently than reported in Phase 3 melanoma clinical trials [6, 7].

When multiple irAEs occur simultaneously (e.g., colitis, pruritus, diabetic ketoacidosis, and hypothyroidism), it may aid in diagnosing sarcoidosis. In these cases, clinicians should also consider the possibility of lymph node and lung metastases as a secondary concern.

In idiopathic sarcoidosis, erythema nodosum is more common in women, while arthritis tends to affect men more frequently. Two-thirds of patients achieve remission within a decade, while the remaining one-third suffer from persistent disease, often leading to significant organ damage. Mortality is low, with fewer than 5% of patients dying from sarcoidosis, typically due to pulmonary fibrosis.

We report differential diagnosis in Table 1 and international guideline recommendations in Table 2.

We reviewed the literature and summarized our findings in Table 3.

Our study describes three melanoma patients who developed sarcoidosis while undergoing immunotherapy or BRAF-targeted treatment. A better understanding of irAEs and enhanced monitoring strategies are crucial for optimizing patient care.

## 2. Case Reports

### 2.1. Patient 1

A 55-year-old female patient visited our outpatient clinic. She had a prior diagnosis of nonmelanoma skin cancer, specifically facial actinic keratosis, which had been treated with 3% diclofenac gel. Her medical history was otherwise unremarkable, except for idiopathic hearing loss in her left ear. In November 2010, the patient underwent excision of a superficial spreading melanoma on her left thigh, classified as pT1a. In November 2020, another atypical nevus was surgically removed from her thigh near the previous melanoma excision site. Histopathological examination showed no evidence of residual melanoma. In July 2022, a nodular melanoma, classified as pT3a, was excised from her left forearm. The Breslow thickness was 2.5 mm, and the tumor had no lymphocytic infiltrate, no ulceration, no pigmentation, and no regression. It exhibited 16 mitoses per square millimeter, with no evidence of vascular, neural invasion, or microsatellitosis.

After a total body CT scan in November, she received radicalization of melanoma in situ and removal of lymph nodes at the elbow level and in the axillary area (sentinel lymph node biopsy (SLNB)) after lymphoscintigraphy. In the sections of previous melanoma, no atypical melanocytic elements were observed, but only dermal fibrosis, oedema, dilated vessels, and phenomena of subcutaneous inflammation.

Pathologic histology was pT3, pN1a (subcapsular lymph node localization of melanoma with a maximum diameter equal to 0.08 mm in lymph nodes 2.2 cm long). Subcapsular nevi inclusions are associated.

For her history of multiple melanomas, the patient underwent genetic counseling, with tests conducted at the National Cancer Center in Genova. The genes assessed included ACD, ATM, BAP1, BRACA1, BRAC2, CDK4, CDKN2A, CHEK, EBF3, GOLM1, MBD4, MC1R, TYR, TYRP1, TERF2IP, OCA2, POLE, POT1, PTEN, SDHA, and SLC45A2, as well as TERF2IP. Additionally, a molecular assessment was performed, targeting point mutations and insertions/deletions in regions of 16 clinically significant genes: ALK (Exons 22 and 23), BRAF (Exons 11 and 15), EGFR (Exons 3, 7, 15, 18, 19, 20, and 21), ERBB2 (Exons 20 and 21), FGFR3 (Exons 7 and 9), HRAS (Exons 2 and 3), IDH1 (Exon 4), IDH2 (Exon 4), KIT (Exons 9, 11, 13, 14, 15, 17, and 18), KRAS (Exons 2, 3, and 4), MET (Introns 13 and 14 and Exons 14 and 19), NRAS (Exons 2, 3, and 4), PDGFRA (Exons 12, 14, and 18), PIK3CA (Exons 10 and 21), RET (Exons 10, 11, 13, 15, and 16), and ROS1 (Exon 38). The test identified a variant, c.183A>C (p. Gln61His), in Exon 3 of the NRAS gene, with an allele frequency of 30.78% (classified as Level II). All other regions investigated were found to be wild types.

In November 2023, the patient initiated treatment with pembrolizumab at a dosage of 200 mg every 21 days. After completing seven cycles, she remained free of significant adverse events, except for iatrogenic hypothyroidism, which was managed with levothyroxine therapy.

A CT scan re-evaluation in late March, including the neck, chest, brain, and abdomen with contrast, showed the following findings: lymphadenopathy in the left and right hilum and paratracheal areas, hilar adenopathies, and small perilymphatic nodules in the upper and middle lung zones.

During this period, the patient also developed small subcutaneous nodules. A skin ultrasound revealed hypoechoic and inhomogeneous subcutaneous nodules, ranging from 3 to 10 mm in size, with irregular margins and no vascularization.

**Figure 1:**
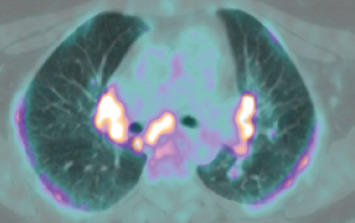
PET-CT body scan before discontinuation of pembrolizumab.

In April, the patient underwent a fluorodeoxyglucose (FDG) PET-CT body scan (Figure 1), which revealed significant findings: intense symmetrical hyperuptake of lymph nodes in the mediastinum, including the upper and lower right paratracheal, precarinal, subcarinal, and aortic arch regions, as well as bilateral pulmonary hilar nodes (the most pronounced uptake was in the subcarinal region, with a maximum standardized uptake value (SUVmax) of 16.70); in both lung fields, there was a predominant subpleural distribution of hyper-uptake, associated with fine diffuse interstitial thickening seen in the coregistered low-dose CT images (with a SUVmax of 8.77 in the left lateral lower lung field); numerous millimetric cutaneous and subcutaneous focal accumulations were noted in both upper limbs, predominantly in the forearms, and in both lower limbs (with the most intense uptake observed anterior to the right patella, SUVmax 11.17). Intense uptake was also seen in the muscles of both legs, particularly in the right tibialis anterior (SUVmax 10.39).

No significant abnormalities were observed in the laboratory findings, including normal calcium levels, absence of lymphopenia, and no elevation of serum angiotensin-converting enzyme (sACE).

**Figure 2:**
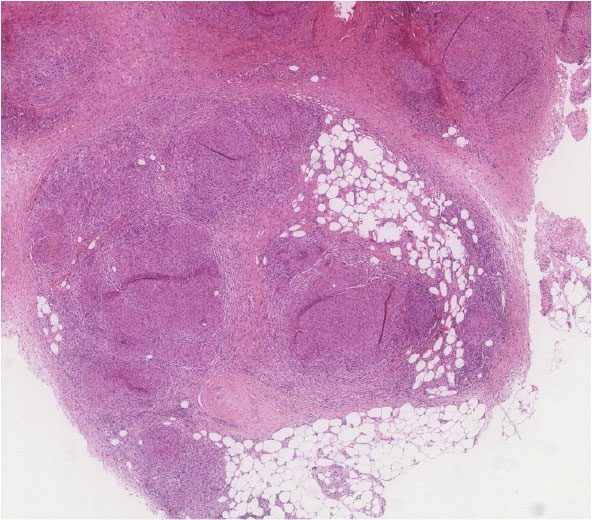
Hematoxylin and eosin, 4x magnification: lobular and septal granulomatous infiltration.

**Figure 3:**
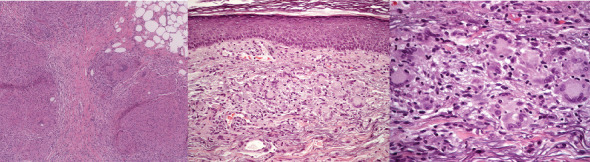
Hematoxylin and eosin, 10x magnification: sarcoid granulomas with foreign body-type giant cells, few lymphocytes, and moderate intragranulomatous fibrosis.

**Figure 4:**
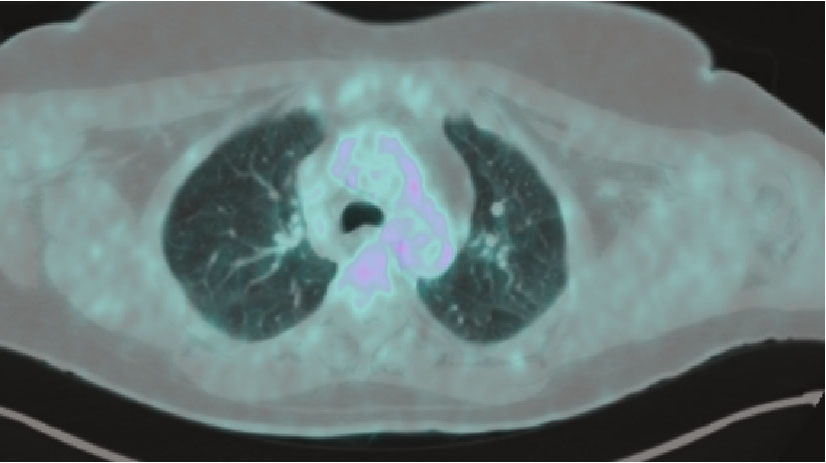
PET-CT body scan response after pembrolizumab suspension.

The patient had subcutaneous nodules excised from the upper left arm and lower left leg. The resected tissue was formalin-fixed, paraffin-embedded, and stained with hematoxylin and eosin. Histological examination revealed that the subcutaneous fat exhibited an extensive granulomatous infiltrate consisting of epithelioid cell granulomas with minimal lymphocytic involvement, consistent with sarcoid granulomas. No melanoma components were detected in the epidermal or dermal layers of the specimens. The granulomatous infiltrates were distributed in a lobular and septal pattern within the subcutaneous fat (Figure 2). Moderate septal and intergranulomatous fibrosis was noted, along with a moderate presence of foreign body-type giant cells (Figure 3). PET-CT scan was repeated at 2 and 8 months from the diagnosis of sarcoidosis and ICI suspension. PET-CT at 8 months (Figure 4) was negative for mediastinal nodes and in general negative for progressive disease regarding melanoma.

In a multidisciplinary team (MDT) discussion, it was recommended to suspend ICI and initiate steroid therapy with prednisone at 1 mg/kg. Angiotensin-converting enzyme (ACE) levels remained normal at 45 U/L, and other parameters such as serum creatinine (1.0 mg/dL), alkaline phosphatase (45 mg/dL), and calcium (10 mg/dL) were within normal ranges. However, vitamin D levels were low.

### 2.2. Patient 2

**Figure 5:**
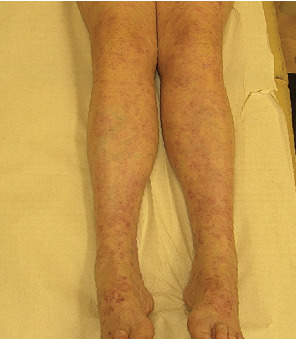
Livedoid macular lesions.

A 48-year-old woman was admitted to our dermatological unit in February 2023 for a generalized cutaneous rash. She was diagnosed with Stage IIIb (T2a, N1b) wild-type melanoma in the left arm and had recently begun adjuvant treatment with nivolumab, administered every 4 weeks at a dose of 480 mg. Seven months after starting ICI, the patient developed a disseminated purplish skin rash with livedoid macular lesions and a vasculitis-like appearance on both upper and lower limbs (Figure 5). There were no other lesions on the skin or visible mucosa. The patient experienced only pruritus (itching) and skin dryness. A biopsy from a lesion on the left lower limb showed multiple nonnecrotizing epithelioid granulomas within the dermis, consistent with sarcoidosis. Additional tests, such as Grocott and Ziehl–Neelsen stains, were done to rule out microorganisms, and other lab results, including complete blood count, serum calcium, protein electrophoresis, ACE, and urinary protein, were within normal limits. QuantiFERON tests and ocular examinations were unremarkable.

Thoracic scans revealed mediastinal lymphadenopathy, suggesting sarcoid lymph node involvement. The major SLR was seen in the skin and subcutaneous tissues. The diagnosis of sarcoidosis was made following the recommendations of Valeyre et al. [22].

The patient's immunotherapy was not discontinued, and the cutaneous lesions and symptoms responded well to treatment with oral methylprednisolone (16 mg/day) and topical clobetasol ointment. The skin lesions began improving after 2 weeks and fully resolved within 2 months. Additionally, the mediastinal lymphadenopathy decreased. As of now, the patient has stable metastatic disease with no progression.

### 2.3. Patient 3

**Figure 6:**
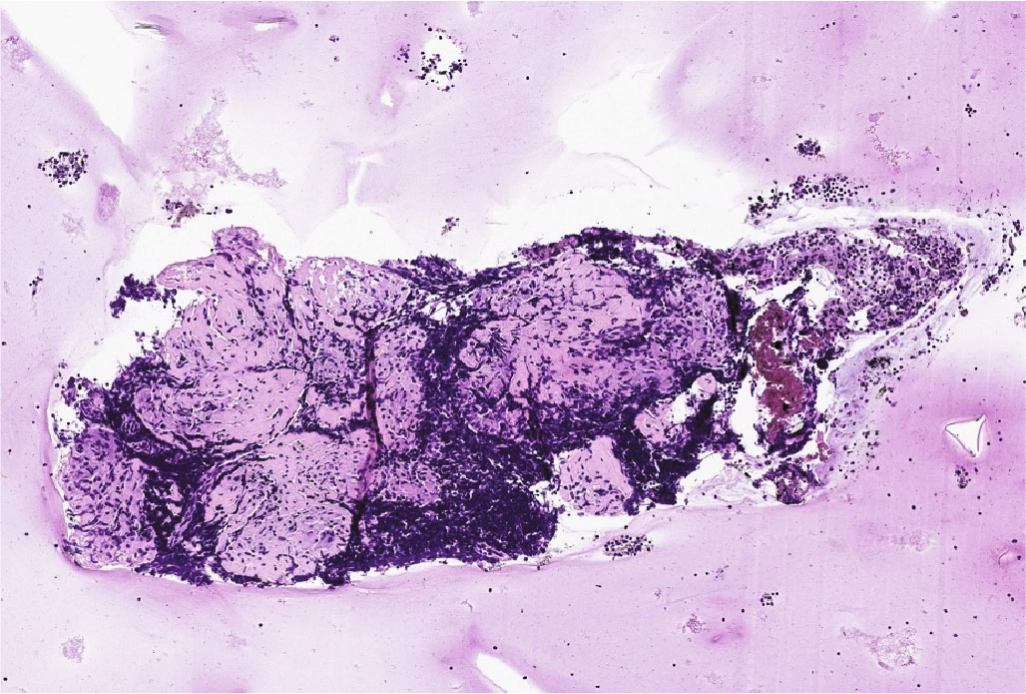
Endobronchial biopsy.

A 65-year-old man, nonsmoker and with no medical history in the past, was admitted to our outpatient department for a pT2N1 melanoma of the trunk. He underwent wide excision and lymph node biopsy, then he was a candidate for an anti-BRAF/MEK inhibitor. Indeed, the tumor was found to have a BRAF V600E mutation. He developed SLR after 142 days after drug administration began. He was admitted to the emergency department for cough, fatigue, and fever; a CT scan showed enlargement of mediastinal nodes. One week of dexamethasone 4 mg die was prescribed. Given the radiographic response, the suspect of progressive disease required further diagnostic evaluation. In the MDT, chest lymphadenopathy was pursued. Endobronchial biopsy of station 7 L lymph nodes demonstrated few polymorphous lymphocyte populations and moderate intragranulomatous fibrosis (Figure 6).

Complete blood count, serum calcium level and urinary calcium level, creatine, protein electrophoresis, alkaline phosphatase, 1,25-OH vitamin D, ACE, and urinary protein level were all within normal limits. QuantiFERON-TB tests and ocular examination did not reveal anomalies. Pneumological function was maintained.

Discontinuation of dabrafenib and trametinib resulted in rapid resolution of symptoms. The patient did not receive any other specific treatment, and after 3 months, the CT scan did not reveal any pathologic imaging. The patient remained off therapy without any recurrence.

## 3. Discussion

Several factors may explain the development of SLRs in patients treated with ICIs, though limited data are available. It has been suggested that modifications in the tumor microenvironment, such as increased levels of TNF-*α*, interferon-gamma, T cell infiltration, and reduced levels of interleukin-6 and interleukin-8, could be associated with irAEs and treatment outcomes.

According to ESMO guidelines, there are no contraindications for restarting therapy, especially in cases where sarcoidosis presents only as extrapulmonary involvement. Several studies have also reported no need for treatment discontinuation under these circumstances. The European Respiratory Society Task Force issued 12 recommendations related to this condition [23].

The link between SLR and targeted therapies like BRAF/MEK inhibitors is less straightforward. Approximately a decade ago, Lheure et al. [12] conducted a retrospective analysis of 70 patients treated with vemurafenib for BRAF-mutant advanced melanoma, finding that five (7.1%) developed SLR, a rate higher than typically expected for melanoma. Since then, several other studies have confirmed the association between anti-BRAF/MEK therapies and the occurrence of SLR.

Several additional studies [12, 24–27] have confirmed the association between BRAF/MEK inhibitors and SLRs, with reported incidences in real-world settings ranging from 3% to 11%. The true rate might be underestimated, as not all patients undergo biopsies, leading to potential misdiagnosis. Accurate diagnosis often requires histological confirmation and multidisciplinary evaluation.

The American Thoracic Society (ATS) recommends using three major criteria for diagnosing sarcoidosis: a compatible clinical presentation, identification of nonnecrotizing granulomatous inflammation in one or more tissue samples, and exclusion of other causes of granulomatous disease. After histological confirmation, the ATS advises baseline testing of serum creatinine, alkaline phosphatase, calcium, and 25- and 1,25-OH vitamin D levels to assess for renal, hepatic, calcium metabolism, and ocular involvement in sarcoidosis [28].

Various studies have highlighted the wide range of presentations and symptoms in melanoma patients who develop sarcoidosis, either at diagnosis or during treatment. Some patients may only exhibit cutaneous lesions, while others may have isolated lung involvement. Sarcoidosis can be diagnosed before melanoma onset or during recurrence. Importantly, the response to sarcoidosis treatments and anticancer therapies can differ, adding complexity to patient management [8, 9].

As reported in various studies, the onset of SLRs appears to be independent of treatment timing, with some patients developing these manifestations even months after completing therapy. In our cohort, two patients developed SLR after seven administrations of different drugs, while the third patient developed it 5 months after starting anti-BRAF/MEK therapy.

Notably, ACE levels, calcium, and alkaline phosphatase levels were normal in all cases. One patient exhibited only skin involvement, while the other two had both skin and pulmonary manifestations. In these two cases, chest CT scans revealed subpleural micronodular opacities, a typical finding in sarcoidosis, along with ground-glass infiltrates, which are less commonly associated with the condition. No eye or renal involvement was observed in any of the patients. Despite ACE levels typically being elevated in sarcoidosis, they remained within the normal range for all three patients. Pulmonary function tests, ECG, and echocardiography were also normal.

Although immune-induced reactions generally have favourable outcomes, the follow-up period in these cases was relatively short, and many reports primarily focus on patients receiving adjuvant treatments. Further long-term data are needed to fully understand the prognosis following SLRs.

## 4. Conclusions

Sarcoidosis and SLRs are characterized by a wide range of cutaneous and extracutaneous manifestations. These can occur with or without symptoms and may present either during or independently of antineoplastic treatments, such as targeted therapies or ICIs. Clinical presentations can vary in severity, from mild to severe. It has been hypothesized that the immune response triggered against melanoma could be a contributing factor in the formation of granulomas. More cases should be collected to generalize our findings.

Accurate diagnosis requires a multidisciplinary approach. Real-world studies indicate that sarcoidosis is not uncommon, with incidence rates higher than those observed in large Phase 3 trials (where it was reported in less than 2% of patients). Sarcoidosis can mimic metastasis, particularly in the skin and mediastinum, leading to potential diagnostic challenges. As a result, biopsies should always be performed to rule out metastatic disease before initiating any antineoplastic treatment. Additionally, recurrent or metastatic melanoma may develop in proximity to areas affected by sarcoidosis, further complicating the diagnostic process.

## Figures and Tables

**Table 1:** Sarcoidosis differential diagnosis.

Mycobacterial infections	Tuberculosis, leprosy
Other infections	Pneumocystis jirovecii pneumonia, histoplasmosis, aspergillosis, blastomycosis, brucellosis
Rheumatic and vascular diseases	Rheumatoid arthritis, Sjögren's syndrome, systemic erythematosus lupus, Wegener's granulomatosis, giant cell arteritis, Kikuchi–Fujimoto syndrome
Paraneoplastic syndrome	Diffuse large B-cell lymphoma (DLBCL), eosinophilic granuloma (histiocytosis X), follicular Lymphoma, Castleman's disease
Occupational exposure	Aluminium, beryllium, zirconium, cobalt, titanium
Drug-induced	BCG, vemurafenib, cobimetinib, dabrafenib trametinib, pembrolizumab, nivolumab, anti-TNF-*α*, methotrexate

**Table 2:** International and national guidelines, definitions, and treatment indications for SLR.

**Guidelines**	**Definition**	**Indications**
ESMO	An SLR is a rare event that involves mainly the lymph nodes, lungs, and skin.	Most reported SLRs are sensitive to CS treatment or discontinuation of ICI. In case of observed benefit from ICI, if the patient is asymptomatic, therapy can be continued. If the patient is symptomatic, then lower doses of CS ≤ 0.5–1 mg/kg can be considered, and ICI therapy can be resumed after the resolution of the irAE.
AIOM	Not defined.	The onset of sarcoidosis or SLR requires suspension of treatment and should be managed as part of a multidisciplinary approach.
ATA	Three major criteria: a compatible clinical presentation, finding nonnecrotizing granulomatous inflammation in one or more tissue samples, and the exclusion of alternative causes of granulomatous disease.	Consider prednisone or methylprednisolone 1–2 mg/kg/day.
ERS	Pulmonary and cutaneous disease.	For untreated patients with major pulmonary sarcoidosis believed to be at higher risk of future mortality or permanent disability from sarcoidosis, we recommend introducing glucocorticoid treatment to improve and/or preserve FVC and QoL. For patients with cutaneous sarcoidosis and cosmetically important active skin lesions that local treatment cannot control, we suggest oral glucocorticoids be considered to reduce skin lesions.

**Table 3:** Sarcoidosis-like reactions induced by checkpoint inhibitors or targeted therapy.

		**Treatment**	**Treatment suspension**	**Sarcoidosis treatment**	**Outcome**
Gouveris et al. [8]	2/133 (1.5%)	1 TT 1 no therapy	Yes	Hydroxychloroquine 200 mg daily, prednisolone 15 mg daily	CR
Dimitriou et al. [9]	8/200	4 ICIs 2TT 2 no therapy	No	2 patients with iv prednisone 2 patients with oral prednisone	PD
Koelzer et al. [10]	1	ICI	Change ICI	iv prednisone	Death
Danlos et al. [11]	1	ICI	Yes	Spontaneous resolution	CR
Lheure et al. [12]	20/1199	7 ICI 13 no therapy	Yes	4/20 steroids	CR
Adam et al. [13]	2	2 TT	No	Hydroxychloroquine 200 mg daily, prednisolone 15 mg daily	CR
Tetzlaff et al. [4]	56 (3)^a^	ICI	38% stopped	44% of patients were treated with systemic steroids and 8% of patients with localized therapy. 96% of patients demonstrated resolution or improvement of granulomatous lesions irrespective of medical intervention	CR
Montaudié et al. [14]	1	ICI	Yes	Oral corticosteroids	CR
Andersen et al. [15]	1	ICI	No	Spontaneous resolution	CR
Murphy et al. [16]	1	ICI	Yes	iv corticosteroids	CR
Reule and North [17]	1	ICI	Yes	iv corticosteroids	PD
Wilgenhof et al. [18]	1	ICI and TT	Yes	iv corticosteroids	PD
Berthod et al. [19]	1	ICI	Yes	Oral prednisone	CD
‍Vogel et al. [20]	1	ICI	Yes	iv corticosteroids	CR
‍Tissot et al. [21]	1	ICI	No	iv corticosteroids	CR

Abbreviations: CR, complete response; ICIs, immune checkpoint inhibitors; iv, intravenous; PD, progressive disease; T, therapy; TT, target therapy.

^a^Single institution.

## Data Availability

The data that support the findings of this study are available from the corresponding author upon reasonable request.
